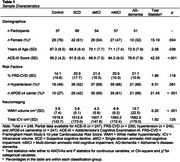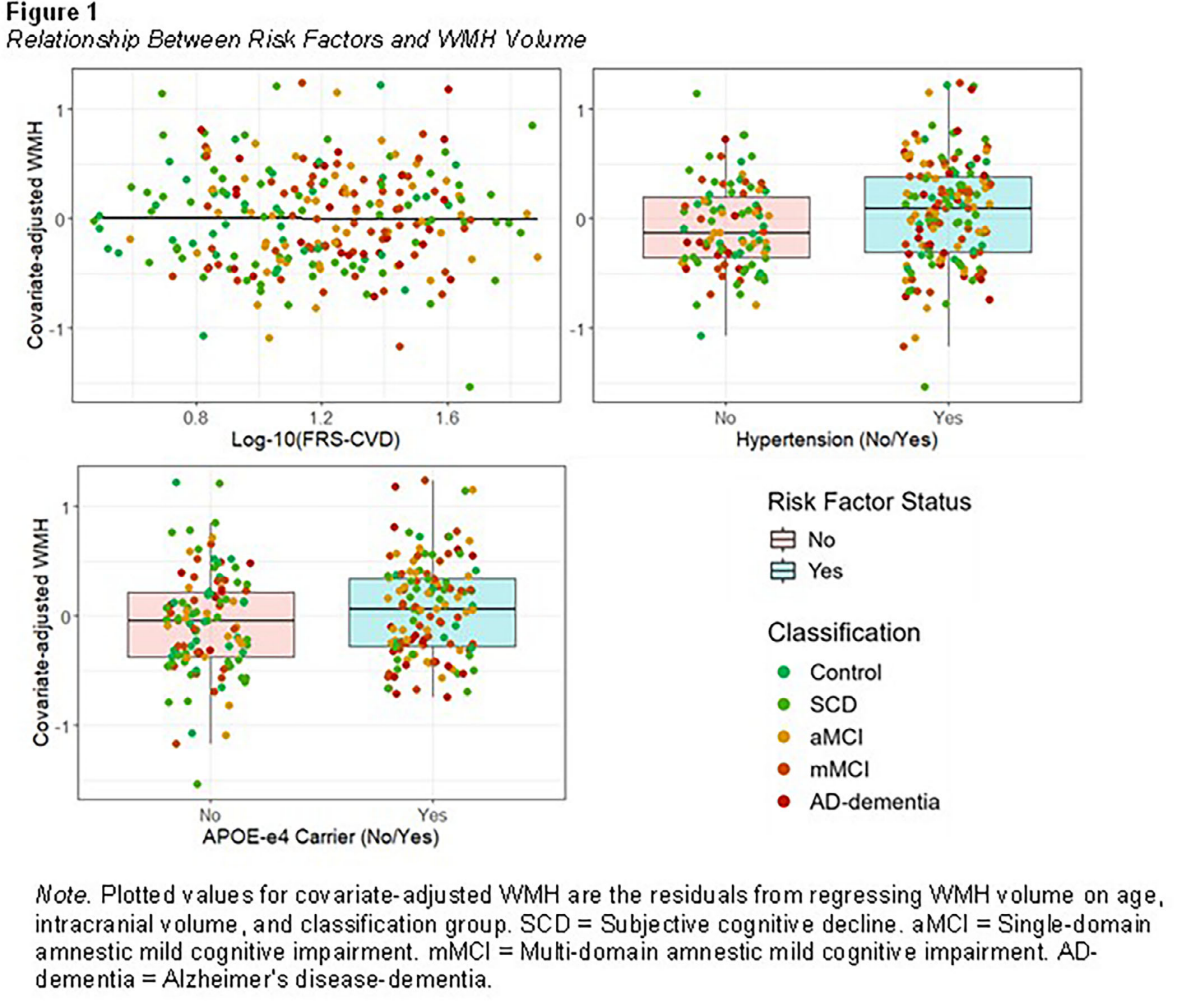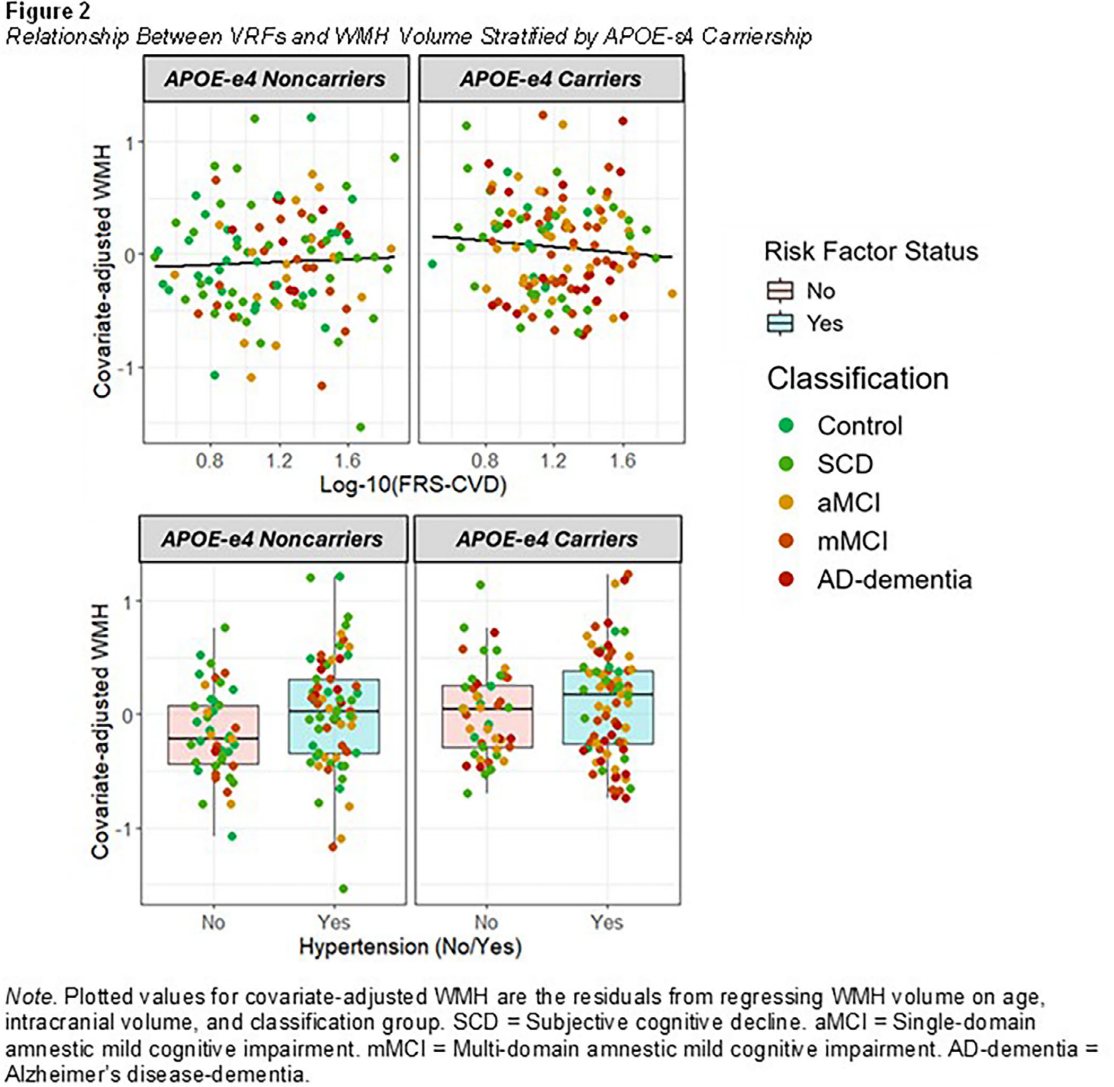# Evaluating Potentially Synergistic Effects of *APOE*‐ε4 and Vascular Risk Factors on White Matter Hyperintensity Burden

**DOI:** 10.1002/alz70856_106032

**Published:** 2026-01-07

**Authors:** Cameron C Heyman, Erin E Cawston, Cameron Stolker, Tracy R Melzer, Campbell J Le Heron, Reece P Roberts, Ian J Kirk, Kiri L Brickell, John C Dalrymple‐Alford, Tim J Anderson, Nicholas J Cutfield, Catherine A Morgan, Lynette J Tippett, the NZ‐DPRC

**Affiliations:** ^1^ School of Psychology, The University of Auckland, Auckland, New Zealand; ^2^ Centre for Brain Research, The University of Auckland, Auckland, New Zealand; ^3^ Department of Pharmacology, University of Auckland, Auckland, New Zealand; ^4^ New Zealand Brain Research Institute, Christchurch, New Zealand; ^5^ Te Kura Mahi ā‐Hirikapo | School of Psychology, Speech and Hearing, University of Canterbury, Christchurch, New Zealand; ^6^ Department of Medicine, University of Otago, Christchurch, New Zealand; ^7^ University of Otago, Christchurch, New Zealand; ^8^ School of Medicine, University of Auckland, Auckland, New Zealand; ^9^ Department of Medicine, University of Otago, Dunedin, New Zealand

## Abstract

**Background:**

White matter hyperintensities (WMHs) observed on MRI are indicative of cerebral small vessel disease. WMHs are prevalent in ageing and might be more severe in the context of Alzheimer's disease (AD). Though genetic and vascular risk factors (VRFs) may play a role, whether these factors directly influence WMH burden is unclear. We investigated whether *APOE‐*ε4 and VRFs were each associated with greater WMH burden and then examined whether associations between VRFs and WMH burden were stronger in *APOE‐*ε4 carriers.

**Method:**

Participants (*n* = 248) from New Zealand Dementia Prevention Research Clinics classified as control, subjective cognitive decline, amnestic single‐ or multiple‐domain mild cognitive impairment, or early AD‐dementia were included. *APOE* genotyping was performed on DNA from fasting bloods. A composite VRF, the Framingham Heart Study's cardiovascular risk score (FRS‐CVD) was calculated. Hypertension was also analysed separately given its associations with increased dementia risk and small vessel changes related to WMHs. Hypertension was considered present if participants either had a history of hypertension, were on hypertension treatment, had systolic blood pressure (BP) ≥140mmHg, or diastolic BP ≥90mmHg. Three multiple linear regression models evaluated whether *APOE‐*ε4 carriership, FRS‐CVD, or hypertension predicted WMH volume. Two multiple regression models tested interactions between *APOE‐*ε4 and each VRF. Covariates in regression models included classification group, age, and intracranial volume (ICV). Standardised regression coefficients (β) and *p*‐values were reported for statistically significant predictors of interest.

**Result:**

WMH volume was greater in *APOE‐*ε4 carriers compared to noncarriers (*p* = .025, β=0.11). WMH volume was also greater in those with hypertension (*p* = .022, β=0.12). Age, ICV, and classification group were also significant predictors of WMH volume. The FRS‐CVD was not associated with WMH volume (*p* = .941, β=‐0.00). *APOE‐*ε4 allele presence did not interact with VRFs in predicting WMH volume (*p*>.05 for *APOE‐*ε4*FRS‐CVD and *APOE‐*ε4*hypertension).

**Conclusion:**

We found *APOE‐*ε4 and hypertension are each associated with greater WMH burden, but that these risk factors do not appear to work synergistically. Whereas hypertension is a strong risk factor for arteriolosclerosis that is associated with WMHs, impaired blood‐brain barrier integrity is linked to the *APOE‐*ε4 allele and might be one mechanism by which *APOE‐*ε4 contributes to WMH burden.